# Evaluating the multi-dimensional values of bridge-tourism integration: an empirical study using the DEMATEL—ISM—MICMAC method

**DOI:** 10.3389/fpubh.2025.1566420

**Published:** 2025-07-17

**Authors:** Yan Du, Han He

**Affiliations:** ^1^Department of Management Engineering, Guizhou Communications Polytechnic University, Guiyang, China; ^2^Department of Chemical and Environmental Engineering, Guizhou Industry Polytechnic College, Guiyang, China

**Keywords:** tourism integration, cultural-ecological-economic values, DEMATEL—ISM—MICMAC method, sustainable tourism, multi-dimensional evaluation

## Abstract

In the context of high-quality tourism development and the increasing emphasis on ecological conservation, bridge-tourism integration has emerged as an innovative model combining cultural, ecological, and economic values, demonstrating significant research potential. However, systematic analysis and comprehensive evaluation of this integration remain limited. To address this gap, the present study applies the integrated Decision-Making Trial and Evaluation Laboratory, Interpretive Structural Modeling, and Cross-Impact Matrix Multiplication Applied to Classification (DEMATEL—ISM—MICMAC) approach to construct a multi-layered evaluation model for assessing these values. The findings identify historical context and architectural design as key drivers, significantly enhancing cultural appeal and ecological value. Intermediate factors, such as cultural activities and geographical location, are essential links within the system. Sub-base factors, including policy regulations and environmental friendliness, provide critical support for cultural preservation and ecological sustainability. In contrast, base-level factors, such as economic benefits and visitor satisfaction, directly reflect the integrated performance of bridge-tourism development. The study concludes that, through systematic management and policy support, bridge-tourism integration can effectively balance cultural preservation, ecological protection, and economic development, laying a solid foundation for its sustainable advancement.

## 1 Introduction

As emblematic structures of human civilization, bridges play a foundational role not only in transportation but also in bearing rich cultural significance and vital ecological value (1). Their design and construction reflect not only advances in engineering and aesthetics but also the social, economic, and cultural contexts of specific historical periods. Thus, bridges are more than physical connectors of space; they serve as carriers of cultural continuity and ecological regulation (2, 3). Against the backdrop of a global shift toward high-quality tourism development, the integration of bridges and tourism—hereafter referred to as *bridge-tourism integration*—has emerged as an innovative tourism model that blends engineering heritage, cultural landscapes, and ecological experiences. This form of tourism demonstrates significant potential in generating multi-dimensional value across cultural, ecological, and economic domains (4).

Although few studies have explicitly examined the integrated value of bridges within tourism systems, existing scholarship across multiple disciplines offers valuable insights that help conceptualize the cultural, ecological, and economic significance of bridges as tourism resources. From the perspective of cultural studies and urban memory, bridges are often regarded as symbolic structures that anchor collective memory and reinforce place identity. Scholars such as Awad, Bakshi, and Broudehoux and Cheli have emphasized how bridges, shrines, monuments, and other urban artifacts function as visual and spatial representations of historical narratives (5–7). These symbols are frequently reinterpreted by different social actors and embedded within urban planning and design, influencing both spatial practices and emotional attachments. Such insights indicate that bridges, beyond their utilitarian roles, are key to fostering cultural resonance and place-based meaning in urban tourism. In tourism research, infrastructure—particularly iconic and walkable forms such as bridges—has been shown to shape destination image, support tourist motivation, and mediate spatial experience (8, 9). For instance, bridges enhance pedestrian accessibility and provide scenic routes, while also serving as landmarks that convey cultural atmosphere. Studies on destination perception suggest that tourists' affective evaluations are strongly tied to environmental aesthetics, accessibility, and infrastructure symbolism, which bridges embody. Environmental planning literature further highlights the ecological value of bridges in maintaining landscape connectivity, reducing fragmentation, and enhancing ecosystem resilience (10–12). Bridges can function as ecological corridors, supporting species movement, mitigating the impacts of urbanization, and contributing to sustainable spatial configurations. However, despite these parallel strands of research, few attempts have been made to integrate these insights into a comprehensive evaluation framework. Studies seldom examine how the cultural, ecological, and economic roles of bridges interact within tourism contexts. This gap underscores the need for a multidimensional approach to assessing bridge-tourism integration, which this study seeks to address.

Southwest China—especially the provinces of Guizhou, Yunnan, and Sichuan—offers an ideal setting for the present study. This region is characterized by rugged karst landscapes and an exceptionally high density of bridges with intricate structures and rich cultural symbolism. Guizhou alone is often referred to as a “living museum of bridges” and is widely recognized in both academic and industry discourse as the epitome of China's bridge-building achievements. The phrase “To understand bridges worldwide, look to China; to understand bridges in China, look to Guizhou” captures this distinction. In such mountainous regions, bridges are not merely infrastructure—they symbolize resilience, community ingenuity, and a spirit of collective perseverance. Accordingly, this study selects the southwestern region of China as its empirical focus, examining typical cases of bridge-tourism integration and analyzing their performance across multiple levels: project, community, and region.

Due to the inherently interdisciplinary nature of bridge-tourism integration, the study draws on a multi-disciplinary framework. It is anchored in tourism management while integrating methodological tools from systems science, place-based perspectives from cultural geography, and assessment approaches from ecological engineering. A three-dimensional evaluation framework is constructed, encompassing cultural, ecological, and economic value dimensions. Methodologically, the study employs a hybrid application of DEMATEL (Decision-Making Trial and Evaluation Laboratory), ISM (Interpretive Structural Modeling), and MICMAC (Cross-Impact Matrix Multiplication Applied to Classification). This integrated approach enables a comprehensive, multi-stage process of factor identification, hierarchical decomposition, and influence classification—providing a structured lens through which to analyze the complexity of bridge-tourism systems.

Therefore, this study utilizes the DEMATEL—ISM—MICMAC approach to construct a system of influencing factors for the multi-dimensional values of bridge-tourism integration, combining structural aesthetics, cultural heritage functions, and ecological protection considerations. In addition, it proposes a systematic evaluation model for integrated performance, contributing both theoretical insights and empirical evidence to inform policy and planning in the pursuit of high-quality tourism development. In summary, this study contributes a novel analytical framework for evaluating bridge-tourism integration as a complex value system. It addresses theoretical gaps in the understanding of value interaction across cultural, ecological, and economic dimensions and provides strategic insights for the sustainable transformation of bridge resources in mountainous regions of Southwest China.

## 2 Literature review

In recent years, the concept of sustainable development has gained global prominence, positioning high-quality tourism development as a central focus of both academic research and practical exploration. Existing studies have primarily concentrated on areas such as the preservation and development of ethnic culture, spatial differentiation analysis, pathways for red tourism, and ecological tourism linked to green transformation. In ethnic culture preservation and development, scholars have emphasized the critical role of protecting and rationally utilizing cultural resources in promoting high-quality tourism. Wang et al. highlighted the significance of toponymic cultural heritage in fostering cultural transmission and tourism (13). However, previous studies have predominantly focused on toponymic words, neglecting cultural entities and environmental factors. This study develops a toponym database, applies a decision tree model to identify relevant heritages, and proposes an evaluation framework that considers internal and external conditions. Tan et al. investigated the impact of the intangible cultural heritage (ICH) listing system on tourism (14). Using data from 2000 to 2019, the study found that regions with more cultural diversity and ICH resources benefit most from ICH listings, leading to a boost in both domestic and international tourism. Regarding spatial differentiation and dynamic evolution, Volgger analyzed the evolution of Airbnb and other peer-to-peer accommodations used by international visitors in Australia between 2015 and 2017. Using dynamic logistic regression, the study examined changes in user characteristics over time. It revealed that Airbnb consumption has shifted toward convergence and normalization, with growing participation from Asian users and an increase in regional stays. This trend, however, was not observed on other platforms, suggesting that peer-to-peer accommodation is transitioning into a single-platform model rather than a diverse category (15). Research on red tourism has focused on its cultural heritage. Zhong and Peng explored how tourism enriches children's experiences, particularly in recreational and red tourism (16). Urban children in China tend to view tourism as a form of play, with recreational tourism as a family activity, while red tourism emphasizes educational values such as patriotism. This study provides insights into children's unique perspectives on tourism, contributing to family tourism practices. Calderón-Fajardo conducted a systematic review of red tourism in China, analyzing its development, impacts, and management strategies (17). Using PRISMA and Bibliometrics, the study identified research gaps and suggested future directions. It highlighted the focus on China and the need for local partnerships and community involvement in red tourism management. In ecological tourism and green transformation, Hao et al. assessed eco-efficiency in the Yellow River Basin using the DPSIR and SBM models, revealing regional disparities and offering policy recommendations for ecological protection (18). Chen et al. examined the integration of sustainable energy in China's Eco-Industrial Parks (EIPs) and its role in promoting green tourism, identifying key challenges and solutions (19). Zhang et al. explored the eco-efficiency of tourism destinations in China, emphasizing the balance between economic and ecological sustainability in the context of urbanization and post-pandemic recovery (20). Despite these advances, bridge-tourism integration—a novel tourism model that embodies cultural and ecological values—remains underexplored in the literature. Current studies predominantly focus on the historical and technical aspects of bridges, with little attention to the multifaceted values of bridge-tourism integration. This gap restricts the full realization of bridge-tourism integration's potential and hinders its strategic application in the development of high-quality tourism.

## 3 Key conceptual definitions

To enhance the scientific rigor of the index system and ensure conceptual clarity throughout the study, this paper defines the three core constructs—cultural value, ecological value, and economic value—within the context of bridge-tourism integration. Their respective connotations and interactive logic are elaborated as follows.

Cultural value refers to the meanings, collective memories, and sense of identity conveyed by bridges as heritage assets and symbolic carriers in the tourism process. It encompasses dimensions such as historical background, architectural aesthetics, and cultural activities (21, 22). This value is reflected not only in tourists' perception of bridge aesthetics and cultural significance, but also in the capacity of bridges to stimulate local cultural vitality and community identity (23). In the context of bridge-tourism integration, cultural value is actualized through mechanisms of heritage preservation, symbolic reproduction, and participatory transmission. Ecological value denotes the capacity of bridges and their surrounding environments to maintain the structural stability and functional integrity of ecosystems during tourism development and operation (11). This includes aspects such as environmental friendliness, ecological education, and the application of sustainable technologies (24). Unlike classical conservationist approaches, this study conceptualizes ecological value through the lens of coordinated regulation within a tripartite relationship among ecosystems, built environments, and human activities (25). The focus lies on minimizing ecological disturbances, enhancing environmental awareness among visitors, and embedding green technologies into tourism experiences (26). Economic value emphasizes the tangible contributions of bridge-tourism integration to regional development, including job creation, consumption stimulation, and income generation (27, 28). Moving beyond traditional financial metrics, this study adopts a broader perspective that highlights the multi-level and sustainable nature of economic value—specifically, the catalytic role of bridges in tourism value chains, the feedback mechanisms that support community economies, and their strategic positioning in regional development frameworks (29, 30). Key indicators include visitor satisfaction, tourism experience quality, and economic returns (31). The term “natural landscape” refers not only to the geomorphological features of the bridge's location—such as mountains, rivers, forests, and wetlands—but also to its relatively undisturbed, native ecological conditions and overall spatial integrity (32). In tourism studies, the appeal of natural landscapes is often attributed to their uniqueness, authenticity, and ecological integrity (33, 34). In bridge-tourism integration, natural landscapes serve both as aesthetic and cultural backdrops and as essential carriers of ecological value. The “local community” refers to the resident population and associated social networks in the area where the bridge is located (35). This concept includes community organization, resident behavior, cultural identity, and local governance structures. In the context of tourism development, local communities are viewed not only as resource providers and service recipients but also as key stakeholders and co-managers (36). This study emphasizes the participatory role of local communities in bridge-tourism integration, focusing on their willingness to engage, cultural expression, and economic benefit (37). Authenticity is a central concept in tourism studies, commonly used to evaluate the perceived genuineness and credibility of cultural resources once they are transformed into tourism products (38). This paper defines authenticity in three dimensions: (1) Cultural authenticity—the preservation of the historical context and symbolic meaning of the bridge without excessive commodification (39). (2) Ecological authenticity—the maintenance of the original ecosystem and environmental integrity in the surrounding landscape (40). (3) Experiential authenticity—the subjective experience of tourists, encompassing depth of engagement, perceived reliability of information, and emotional resonance (41).

## 4 Integrated method and indicator system

### 4.1 DEMATEL—ISM—MICMAC integrated method

The DEMATEL (Decision Making Trial and Evaluation Laboratory) method combines graph theory and matrix operations to analyze, quantitatively, the interactions between factors within a system, identifying causal relationships and their intensity (42). Specifically, DEMATEL first constructs a direct influence matrix that quantifies the strength and direction of relationships among factors, providing a basis for identifying their relative importance and causal roles within the system. These indicators not only evaluate the importance and influence of factors within the system but also provide a quantitative foundation for subsequent hierarchical and driving-force analyses. However, DEMATEL has limitations in structuring hierarchical levels, as it does not categorize the factors within the system into distinct levels. To address this limitation, the Interpretive Structural Modeling (ISM) method is introduced. ISM constructs a directed hierarchical diagram that systematically displays the hierarchical relationships and causal chains between factors. When combined with DEMATEL's results, ISM helps categorize factors based on their causal relationships and hierarchy, thus creating a multi-level framework. This approach not only clarifies the system's structure and enhances logical rigor but also aids in identifying the specific paths of influence at each level (43). However, ISM has limitations in recognizing driving and dependent factors and is less effective at fully capturing the intensity of interactions and dependencies between factors. To further refine the primary and secondary relationships within the same level and their influence intensity, the MICMAC (Matrix Impact Cross-Reference Multiplication) method is introduced. MICMAC calculates the driving power and dependence of each factor, and classifies them into four categories: dependent, autonomous, linkage, and independent factors. By analyzing driving power and dependence in detail, MICMAC refines the primary-secondary relationships within each level, complementing and optimizing the hierarchical structure of ISM. This refinement makes the system structure clearer and more distinct, facilitating the construction and optimization of scientific systems (44).

In the tourism industry and other sectors, the DEMATEL—ISM—MICMAC integrated method has been widely employed for key factor analysis and system structure building (45). For instance, Liu et al. applied this method to study the carbon footprint of prefabricated buildings, revealing the significant impact of material production and transportation on carbon emissions. Alqershy and Shi used the method to analyze the barriers to social responsibility implementation in Belt and Road infrastructure projects, highlighting the constraining effects of cross-national cultural and institutional differences (46). These studies illustrate the significant advantages of the DEMATEL—ISM—MICMAC method in uncovering the multi-level structure and causal relationships of complex systems. However, its application to the analysis of the multiple values of bridge-tourism integration has not been thoroughly explored.

### 4.2 Research steps

***Step 1*:** Constructing the influence factor system

Identify and establish the key factors that contribute to the multiple values of bridge-tourism integration, encompassing cultural, ecological, and economic dimensions. This framework serves as the foundation for subsequent analysis.

***Step 2*:** Conducting expert surveys

Design a questionnaire to evaluate the influence of each factor. Experts are invited to rate the degree of influence on a scale from 0 to 4, where: 4 indicates extremely high influence, 3 indicates high influence, 2 indicates moderate influence, 1 indicates low influence, and 0 indicates no influence. Experts from relevant fields, including bridge design, tourism, ecology, and cultural studies, are selected to ensure diverse and authoritative insights.

***Step 3*:** Constructing the initial direct influence matrix (*B*)

Develop the initial direct influence matrix *B*, defined as *B* = [*b*_*ij*_]_m × *n*_, where: *b*_*ij*_ represents the degree of influence of factor i on factor j, as rated by the experts. The diagonal elements (*b*_*ij*_) represent the self-influence of each factor, which is set to 0 by definition.

***Step 4*:** Determine the standardized direct influence matrix *F* and the comprehensive influence matrix *H*. Where *E* is the identity matrix:
  (1) $$\begin{array}{l} F=B\cdot c \end{array}$$
  (2) $$\begin{array}{l} c=max\left(\frac{1}{m}\overset{m}{\underset{i=1}{\sum }}b_{ij},\frac{1}{n}\overset{n}{\underset{j=1}{\sum }}b_{ij}\right) \end{array}$$
  (3) $$\begin{array}{l} H=F\cdot {\left(E-F\right)}^{-1} \end{array}$$
***Step 5*:** Calculate the influence degree *r*_*i*_ and being influenced degree *c*_*i*_ for each factor:
  (4) $$\begin{array}{l} r_{i}=\overset{n}{\underset{j=1}{\sum }}t_{ij},\;i=1,2,\ldots ,n \end{array}$$
  (5) $$\begin{array}{l} c_{i}=\overset{n}{\underset{i=1}{\sum }}t_{ij},\;i=1,2,\ldots ,n \end{array}$$
***Step 6*:** Calculate the centrality *Z*_*i*_ and causality degree *D*_*i*_ for each factor:
  (6) $$\begin{array}{l} Z_{i}=r_{i}+c_{i},\;i=1,2,\ldots ,n \end{array}$$
  (7) $$\begin{array}{l} D_{i}=r_{i}-c_{i},\;i=1,2,\ldots ,n \end{array}$$
  Where, if causality degree *D*_*i*_ > 0, the factor *f*_i_ is considered a cause factor; if *D*_*i*_ < 0, the factor *f*_i_ is considered an effect factor.
***Step 7*:** Based on the comprehensive influence matrix *H*, determine the threshold γ, and construct the adjacency matrix *T*:
  (8) $$t_{ij}=\{\begin{array}{cc} 1, & h_{ij}\geq \gamma \\ 0, & h_{ij}<\gamma \end{array}$$
  (9) $$\begin{array}{l} \gamma =\bar{x}+\sigma \end{array}$$
  Where $\bar{x}$ is the average value of the factors in matrix *H*, and σ is the standard deviation. This value can simplify the structure and increase the independence between factors.
***Step 8*:** Determine the reachability matrix *K*:
  (10) $$\begin{array}{l} K={\left(T+E\right)}^{n+1}={\left(T+E\right)}^{n}\neq {\left(T+E\right)}^{n-1}\neq \left(T+E\right) \end{array}$$
***Step 9*:** Determine the reduced node matrix *K*′ and the reduced edge matrix *S*′. By merging factors in the horizontal and vertical rows with the same influence relationships, the edge reduction method is as follows:
  (11) $$\begin{array}{l} S^{\prime }=K^{\prime }-{\left(K^{\prime }-E\right)}^{2}-E \end{array}$$
***Step 10*:** Construct the general skeleton matrix *S*. Based on the reduced edge matrix *S*′, replace the loops and obtain the general skeleton matrix *S*.
***Step 11*:** Divide the ISM model into hierarchical levels. Based on the general skeleton matrix *S*, calculate the reachability set *R*(*s*_*i*_), antecedent set *A*(*s*_*i*_), and intersection set *C*(*s*_*i*_):
  (12) $$\begin{array}{l} R\left(s_{i}\right)=\left\{s_{i}\in S|s_{ij}=1\right\}\; \end{array}$$
  (13) $$\begin{array}{l} A\left(s_{i}\right)=\left\{s_{i}\in S|s_{ji}=1\right\}\; \end{array}$$
  (14) $$\begin{array}{l} C\left(s_{i}\right)=R\left(s_{i}\right)\cap A\left(s_{i}\right)\; \end{array}$$
  (15) $$\begin{array}{l} U\left(s_{i}\right)=\left\{s_{i}\in S|R\left(s_{i}\right)\cap C\left(s_{i}\right)\right\}\; \end{array}$$
  (16) $$\begin{array}{l} D\left(s_{i}\right)=\left\{s_{i}\in S|A\left(s_{i}\right)\cap C\left(s_{i}\right)\right\}\; \end{array}$$
***Step 12*:** In the MICMAC model, calculate the driving power *P* and dependence *J*. Based on the reachability matrix *K*, calculate the driving power *P*_*i*_ and dependence *J*_*i*_, and construct the driving power-dependence matrix:
  (17) $$\begin{array}{l} P_{i}=\overset{n+1}{\underset{j=1}{\sum }}k_{ij}\; \end{array}$$
  (18) $$\begin{array}{l} J_{i}=\overset{n+1}{\underset{i=1}{\sum }}k_{ij}\; \end{array}$$

### 4.3 Indicators construction

Systems theory posits that all phenomena are composed of interrelated and interacting elements that form an integrated whole. Only by adopting a holistic perspective can the essential characteristics and operational mechanisms of complex systems be effectively understood (47, 48). From the standpoint of complex systems theory, any entity is conceptualized as a dynamic structure comprised of multiple mutually dependent and interacting components. As such, holistic analysis and relational reasoning are indispensable for uncovering the inner logic of systemic behavior and performance.

Grounded in the foundational principles of systems theory, this study conceptualizes the multiple performance dimensions of bridge-tourism integration as a complex system comprising three interrelated components: the driving dimension, the environmental dimension, and the carrying dimension. The coupling and dynamic interaction among these three dimensions are considered determinative to the realization and optimization of integrated performance.

The application of systems theory in tourism research has gained increasing scholarly attention, particularly in the construction of evaluation index systems. For example, Xue introduced systems theory into the development of a risk assessment framework for adventure tourism, integrating the Delphi method and Analytic Hierarchy Process (AHP) to construct an indicator model grounded in human factors theory and accident causation logic, aimed at improving hiking safety (49). Similarly, Kalipci combined systems theory with resource dependence theory to analyze the transformative effects of e-commerce on tourism operations, highlighting systemic influences across management, modeling, customer satisfaction, and service quality (50). Ko applied systems theory and space syntax in the optimization of tourism signage systems, revealing the influence of spatial configuration on visitor mobility and satisfaction (51). Gan and Liu constructed a systems-theoretic framework for cruise tourism supply chain risk identification, using catastrophe theory to identify key vulnerabilities and enhance operational resilience (52). Zhao and Li proposed a systems model for high-quality enterprise development in the context of digital transformation, identifying an inverted U-shaped relationship between digital intensity and firm performance (53).

In the realm of tourist behavior, Lindberg and Stemmer employed systems theory in conjunction with dual-process cognitive models to explore the development of System 2 decision-making styles, offering new insights into the cognitive foundations of tourism choices (54). Wu et al. built a social-ecological systems resilience framework for coastal tourism destinations in the Beibu Gulf of Guangxi, demonstrating the value of systems theory in sustainable development assessments (55). Moreover, Wang and Fu developed a regional tourism performance evaluation method that integrates fuzzy AHP and radial basis function neural networks, thereby expanding the methodological scope of systems theory in multi-variable performance analysis (56).

#### 4.3.1 Driving dimension

The driving dimension serves as the central force behind the cultural and ecological values of bridge-tourism integration, primarily comprising cultural and ecological factors. Cultural factors include the historical background of the bridge, its architectural design, and associated cultural activities (57). Specifically, the historical background imbues the bridge with rich cultural significance, reflecting the social, economic, and technological characteristics of a particular historical period. The architectural design showcases distinct aesthetic and engineering qualities, enhancing the bridge's visual appeal and cultural value. Cultural activities, such as exhibitions, performances, and festivals held on or around the bridge, foster visitor engagement and enrich their experiences (58). These cultural factors, through multi-dimensional interactions, cultivate a distinctive cultural atmosphere in bridge-tourism integration, facilitating the deep integration of cultural heritage with tourist experiences. Ecological factors encompass environmental sustainability, ecological education, and sustainable technologies (59). Environmental sustainability refers to the impact of the bridge's construction and operation on the ecosystem, emphasizing the importance of minimizing environmental harm (60). Ecological education reflects the bridge's role in raising public awareness of ecological issues and promoting environmental protection concepts. The application of sustainable technologies in the design and construction of the bridge, such as the use of eco-friendly materials and innovative construction methods, supports sustainable development practices. These ecological factors work synergistically to ensure that bridge-tourism integration maintains a balance between ecological conservation and resource sustainability, thereby contributing to the long-term enhancement and preservation of environmental value (61).

#### 4.3.2 Environmental dimension

The environmental dimension provides the essential conditions for the realization of the driving dimension, encompassing both the natural and human environments. The natural environment includes the geographical location of the bridge and the integrity of the surrounding ecosystem, both of which directly influence the tourism appeal of the bridge. A favorable location and a diverse, well-preserved ecosystem enhance the aesthetic and ecological value of the bridge. In contrast, the human environment has policies, regulations, social culture, and infrastructure (62). Policy and legal frameworks offer institutional support for the development of bridge-tourism integration. A strong social and cultural atmosphere, along with the active participation of local communities, enhances the cultural richness and quality of the tourism experience. Well-developed infrastructure, including transportation networks, accommodation, and dining facilities, directly impacts visitor satisfaction and tourism experience (63, 64). The environmental dimension ensures the effective functioning of the driving dimension by providing the necessary support and constraints. It creates a harmonious environment in which bridge-tourism integration can thrive within both the natural and social contexts. The favorable natural environment and robust human environment collectively form the external conditions that facilitate the achievement of tourism value in bridge-tourism integration.

#### 4.3.3 Carrying dimension

The carrying dimension represents the recipient and expression of the value generated through bridge-tourism integration. It primarily includes tourists, local communities, and managers. The tourism experience and satisfaction of visitors directly reflect the effectiveness of bridge-tourism integration, serving as crucial indicators for evaluating its value. By engaging in cultural and ecological activities, tourists experience the multifaceted values resulting from bridge-tourism integration, which influences their overall satisfaction and perception of the tourism project (65, 66). Local communities benefit economically from bridge-tourism integration and gain opportunities to participate in cultural preservation, thus strengthening community cohesion and fostering a sense of identity. The active involvement of local communities not only promotes economic development but also deepens the transmission of cultural heritage, facilitating positive interactions between the community and the tourism project (67). Managers play a critical role in the planning, operation, and long-term development of bridge-tourism integration. Their management expertise and awareness of sustainable development directly impact the quality and effectiveness of the entire tourism project (68). Through scientific planning and efficient management, they ensure the standardization and sustainability of tourism activities, thereby enhancing the operational efficiency and service quality of bridge-tourism integration as a whole (69).

#### 4.3.4 Coupling relationships

**Figure 1:**
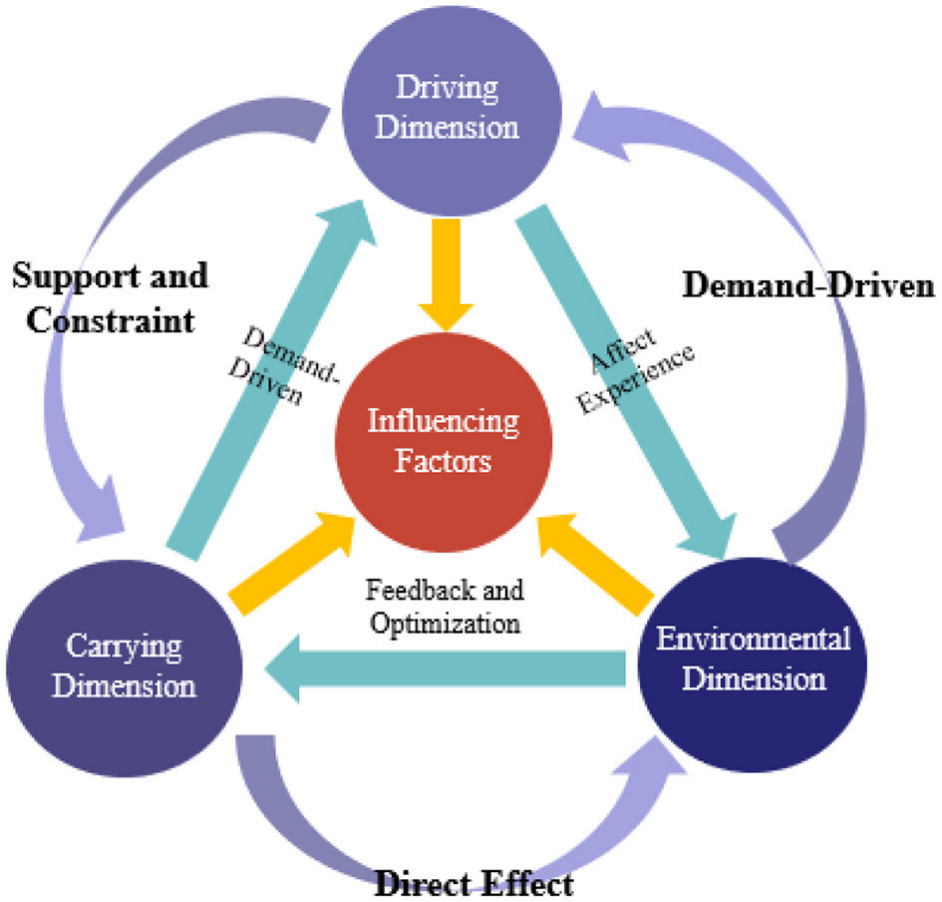
Influence factor model.

The coupling relationships between these three dimensions are depicted in Figure 1. The driving dimension, influenced by the ecological dimension, supports and constrains the carrying dimension. In turn, feedback from the environmental dimension affects the performance of the other two dimensions and fosters their optimization. For instance, the historical and cultural value of the bridge can only be fully appreciated by tourists when there is robust policy support and a positive social and cultural atmosphere. Visitor satisfaction and feedback can prompt managers to refine operational strategies, optimize the environmental dimension, and enhance the overall value of bridge-tourism integration. This dynamic coupling relationship ensures that the bridge-tourism integration system continually adapts to changes in the external environment and internal requirements, thereby promoting the coordinated advancement of cultural heritage, ecological protection, and economic development.

Based on the preceding analysis, this study identifies 17 key influencing factors that shape the multiple values of bridge-tourism integration, which are detailed in Table 1.

**Table 1:** List of key influencing factors for bridge-tourism integration's multiple values.

**Major category**	**Secondary category**	**Influencing factor**	**Description**
Driving dimension	Cultural factors	Historical background (*X*_1_)	Historical origins and cultural significance of the bridge
		Architectural art (*X*_2_)	Aesthetic and engineering design of the bridge
		Cultural activities (*X*_3_)	Cultural events held on or around the bridge
	Ecological factors	Environmental friendliness (*X*_4_)	Environmental impact of the bridge's construction and operation
		Ecological education (*X*_5_)	Role of the bridge in promoting environmental awareness
		Sustainable technologies (*X*_6_)	Use of eco-friendly materials and construction techniques
Environmental dimension	Natural environment	Geographical location (*X*_7_)	Location and landscape characteristics of the bridge
		Ecosystem (*X*_8_)	Integrity and biodiversity of the surrounding ecosystem
	Human environment	Policy and regulations (*X*_9_)	Relevant policies and regulations supporting bridge-tourism integration
		Social culture (*X*_10_)	Cultural atmosphere and local community involvement
		Infrastructure (*X*_11_)	Transportation and service facilities supporting bridge-tourism integration
Carrying dimension	Tourists	Tourism experience (*X*_12_)	Visitors' engagement and experiences within bridge-tourism integration
		Satisfaction (*X*_13_)	Recognition of cultural and ecological values by tourists
	Local communities	Economic benefit (*X*_14_)	Economic benefits from bridge-tourism integration for the local community
		Cultural heritage (*X*_15_)	Local community participation in cultural activities and preservation
	Managers	Management level (*X*_16_)	Planning and maintenance of bridge-tourism integration resources
		Sustainable development awareness (*X*_17_)	Awareness of ecological and cultural protection in management

## 5 Data calculation and analysis

### 5.1 DEMATEL calculation and analysis

In analyzing the mechanisms influencing the multidimensional performance of bridge-tourism integration, this study employed an expert survey method to obtain the direct influence relationships among key variables. The questionnaire respondents were professionals from Southwest China—including Guizhou, Yunnan, and Sichuan provinces—who have long been engaged in fields such as bridge design, cultural tourism planning, ecological and environmental protection, chemical and materials engineering, and policy management. The sample encompassed a broad interdisciplinary representation, including university faculty members, postgraduate students (master's and doctoral levels), industry engineers, and personnel from local government departments, thereby ensuring both academic and practical expertise. In terms of sample composition, academic respondents were primarily affiliated with Guizhou University, Southwest University, and Yunnan University, from which 21 valid questionnaires were collected. Industry and governmental respondents were mainly drawn from organizations involved in bridge construction, cultural tourism, environmental technologies, and government administration. These participants worked in areas such as project planning, engineering implementation, cultural-tourism integration, and industrial investment promotion, yielding 104 valid questionnaires. In total, 130 questionnaires were distributed, and 118 valid responses were obtained after eliminating incomplete or inconsistent entries, resulting in a valid response rate of 90.8%.

The questionnaire adopted a five-point Likert scale (0 to 4) to evaluate the degree of direct influence among 17 key factors, based on expert judgment regarding the coupling relationships between these factors from both cognitive and practical perspectives. To assess the reliability and internal consistency of the collected data, Cronbach's alpha coefficient analysis was conducted using SPSS version 27. Among the 118 responses, 113 questionnaires had alpha coefficients above the acceptable threshold of 0.7, while 5 fell below. This indicates a high level of overall reliability in the survey results. The 113 qualified responses were then subjected to weighted averaging to generate a composite data set for further analysis. A second reliability test on the aggregated dataset yielded a Cronbach's alpha of 0.849, confirming the robustness and consistency of the questionnaire data and its suitability for subsequent modeling and structural analysis.

**Figure 2:**
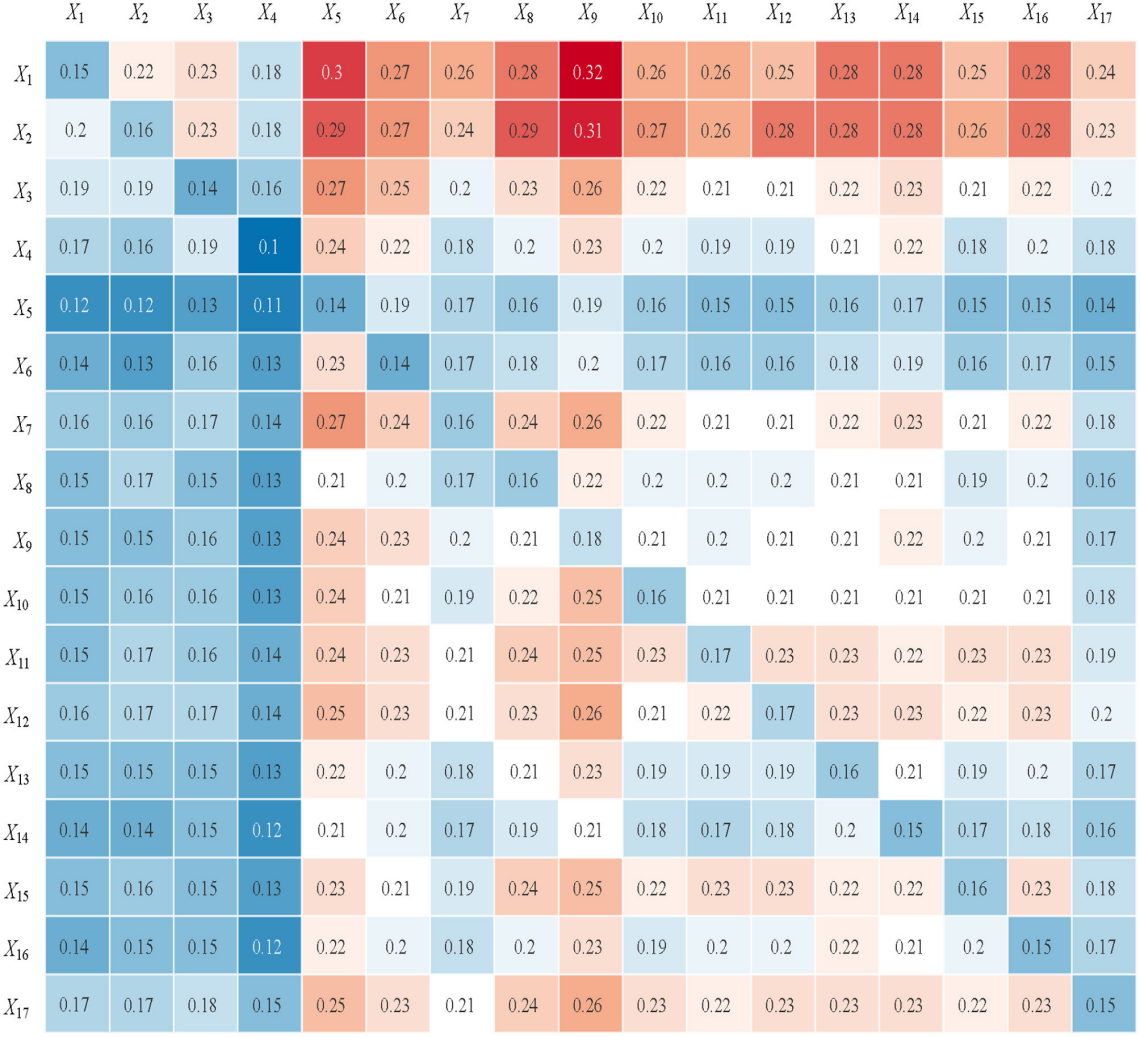
Comprehensive impact matrix *H*.

**Figure 3:**
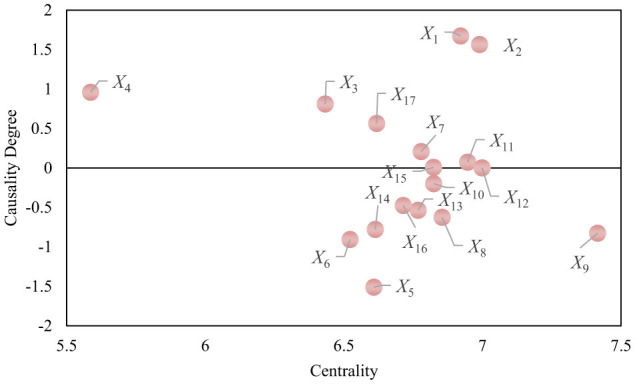
Causal scatter plot of influencing factors.

By calculating the average scores, the initial direct impact matrix *B* was obtained. Subsequently, the comprehensive impact matrix *H* was derived using Equations 1–3, as shown in Figure 2. Using Equations 4–7, the impact degree *r*, affected degree *c*, centrality *D*, and causality *Z* for each influencing factor were computed. The results are presented in Table 2 and Figure 3. A higher centrality value indicates a factor's greater importance within the system, while a larger causality value reflects its stronger influence on other factors. Based on the causality, factors with *Z* > 0 are classified as cause factors, while those with *Z* < 0 are classified as effect factors.

**Table 2:** Influence degree, impact degree, centrality, causality, and factor attributes.

**Factor**	***c_*i*_***	***r_*i*_***	***D_*i*_***	***Z_*i*_***	**Factor attributes**
*X*_1_	2.6245	4.2967	6.9212	1.6722	Causal factor
*X*_2_	2.7127	4.2768	6.9895	1.5641	Causal factor
*X*_3_	2.8112	3.6215	6.4327	0.8103	Causal factor
*X*_4_	2.3137	3.2725	5.5862	0.9588	Causal factor
*X*_5_	4.0594	2.5488	6.6082	−1.5106	Resultant factor
*X*_6_	3.7143	2.808	6.5223	−0.9063	Resultant factor
*X*_7_	3.2855	3.4933	6.7788	0.2078	Causal factor
*X*_8_	3.7398	3.1147	6.8545	−0.6251	Resultant factor
*X*_9_	4.1211	3.2943	7.4154	−0.8268	Resultant factor
*X*_10_	3.5116	3.3124	6.824	−0.1992	Resultant factor
*X*_11_	3.4368	3.5097	6.9465	0.0729	Causal factor
*X*_12_	3.4988	3.4999	6.9987	−0.0011	Resultant factor
*X*_13_	3.6521	3.1158	6.7679	−0.5363	Resultant factor
*X*_14_	3.6961	2.9172	6.6133	−0.7789	Resultant factor
*X*_15_	3.4079	3.4157	6.8236	0.0078	Causal factor
*X*_16_	3.5948	3.1194	6.7142	−0.4754	Resultant factor
*X*_17_	3.0273	3.5909	6.6182	0.5636	Causal factor

As shown in Table 1 and Figure 3, this study quantifies and elucidates the causal relationships and interactions among the influencing factors within the bridge tourism system. It identifies the core driving factors and outcome factors, thereby providing a scientific basis for multi-level value evaluation of bridge tourism. Based on the positive or negative values of causality, the factors are categorized as “cause factors” and “effect factors,” with their significance further assessed through their centrality within the system.

In accordance with system theory, the core driving role of the cause factors within the system is first confirmed. Factors such as historical background (*X*_1_), architectural art (*X*_2_), cultural activities (*X*_3_), environmental friendliness (*X*_4_), and awareness of sustainable development (*X*_17_)—which are positioned above the causality line at *y* = 0—demonstrate substantial driving power. These factors not only exert direct influence within the system but also induce changes in other factors through multi-level causal chains. This driving power corresponds to the “core driving layer” in system theory, wherein the cultural, ecological characteristics, and management awareness of bridge tourism collectively form the foundational elements for the system's operation.

Subsequently, the outcome factors reflect the final performance of bridge tourism, including tourist experience (*X*_12_), economic benefits (*X*_14_), and cultural heritage (*X*_15_). These factors, situated below the causality line at *y* = 0, are primarily influenced indirectly by the driving factors. Their performance serves as a direct reflection of the overall effectiveness of the bridge tourism system across cultural, ecological, and economic dimensions, and is indicative of high-quality development outcomes.

From the perspective of system holism, this analysis reveals the hierarchical structure and causal transmission paths of the various factors within the bridge tourism system. This structure aligns with the multi-level transmission model in system theory, demonstrating that the cultural, ecological, and economic values of bridge tourism do not operate in isolation, but are realized through interconnected causal chains that foster collaborative development. Specifically, the multidimensional linkage of the “cultural-ecological-economic” values reflects the division of labor and cooperation among factors at different levels within the system, offering a theoretical foundation for formulating management strategies. This suggests that priority should be given to intervening in the core driving factors to optimize and regulate the operation of the entire system.

### 5.2 ISM calculation and analysis

**Figure 4:**
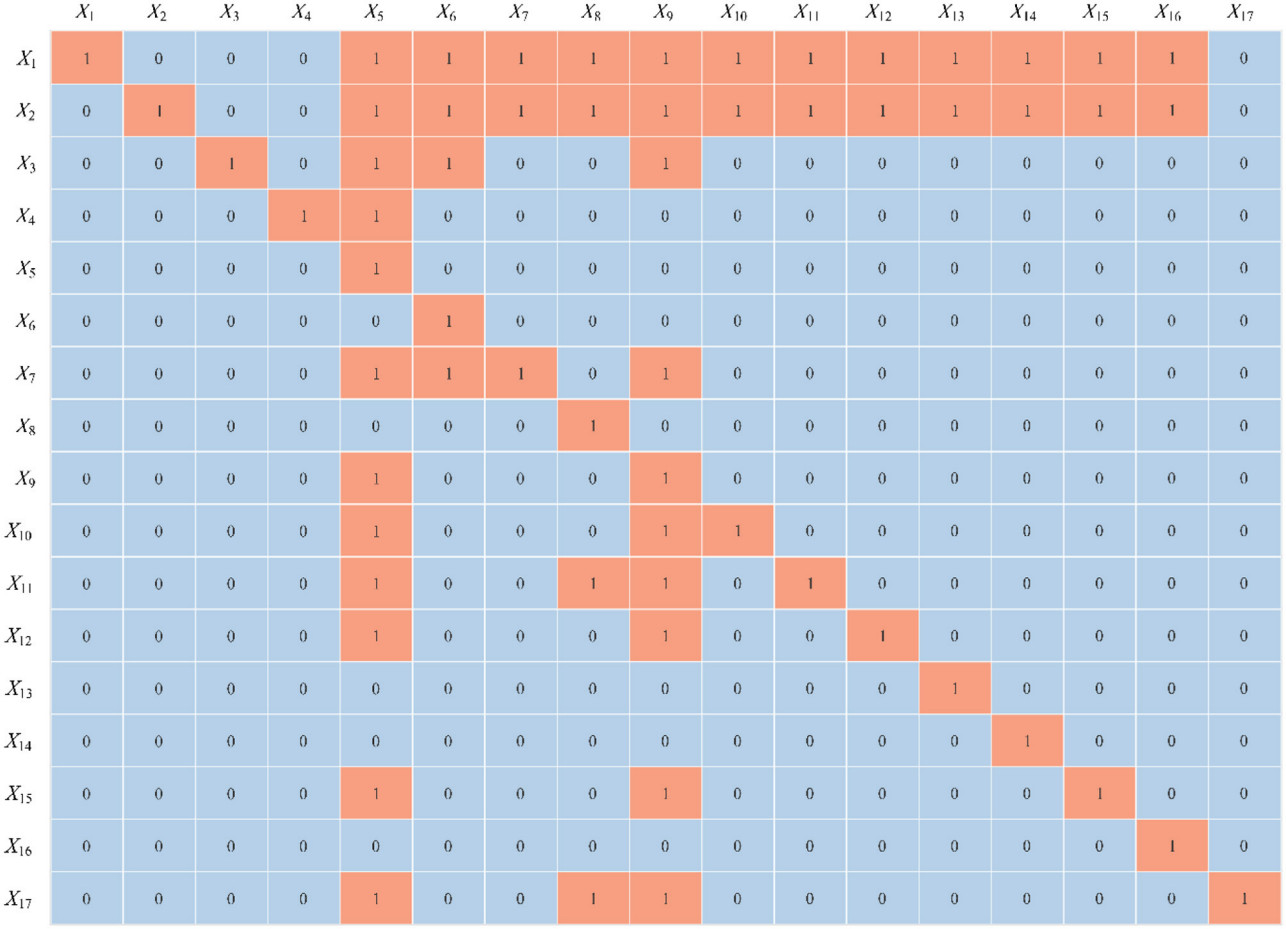
Reachability matrix *K*.

**Figure 5:**
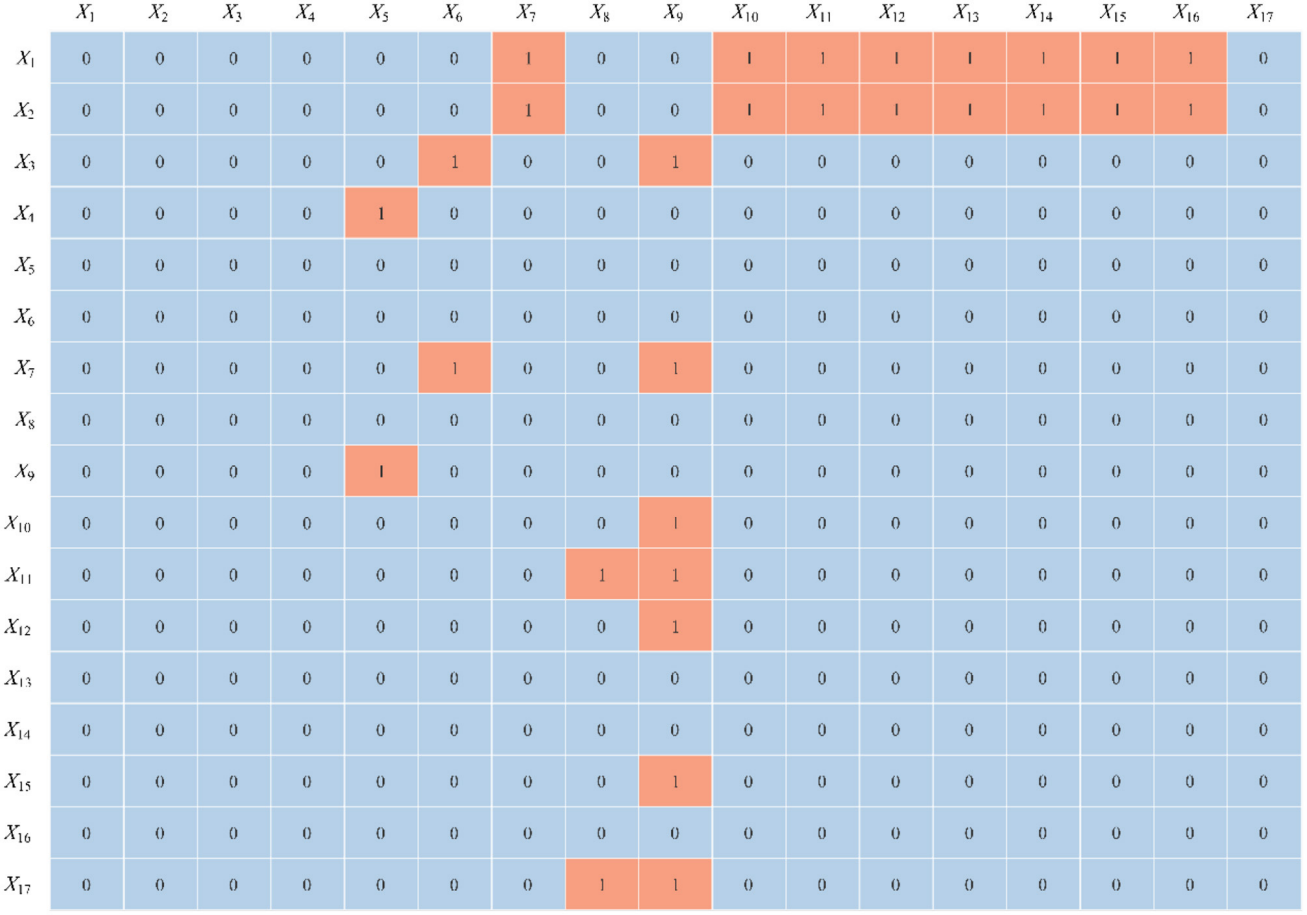
Skeleton matrix *S*.

**Figure 6:**
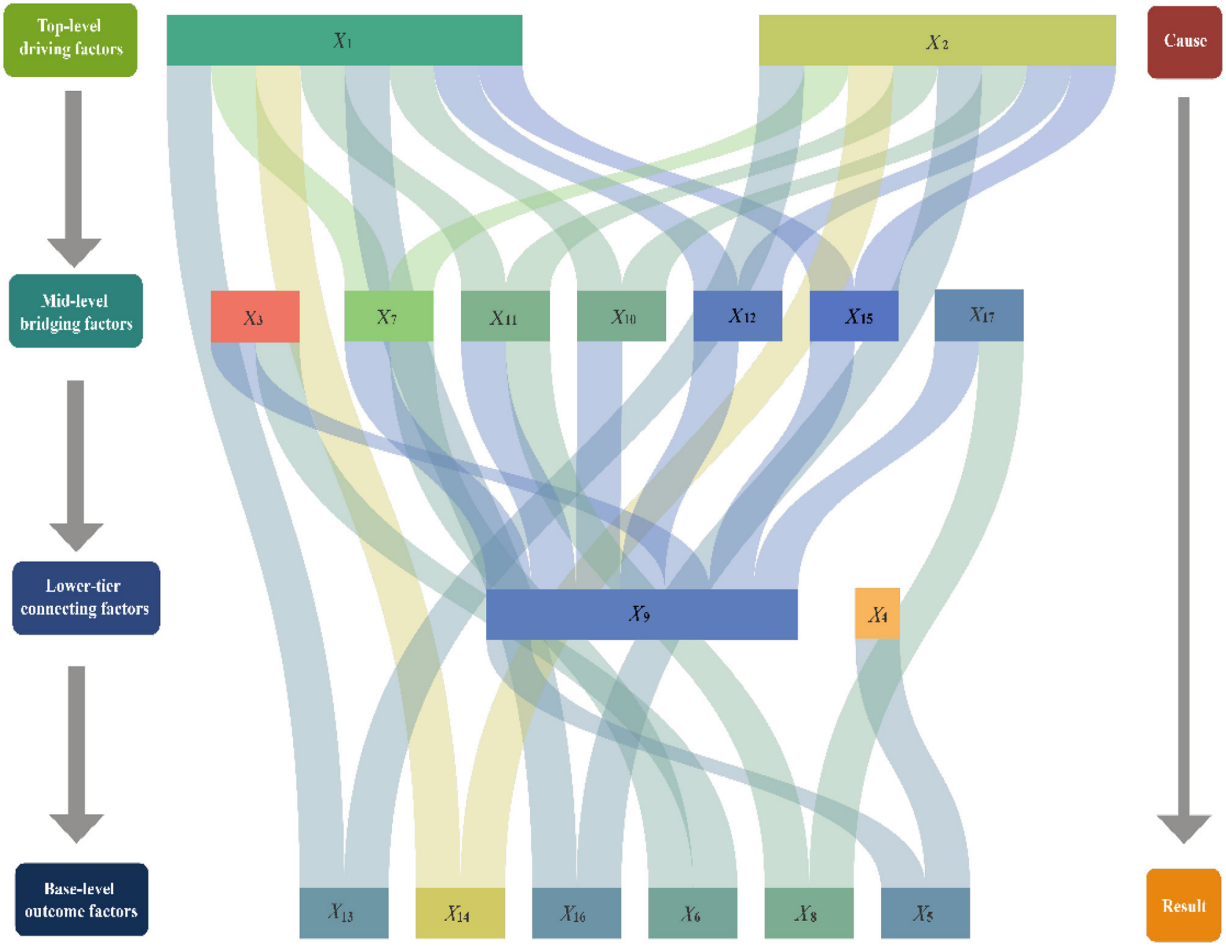
ISM hierarchical partitioning model.

Using Equations 8, 9, the adjacency matrix *T* was computed with a threshold value of λ = 0.1964. Subsequently, the reachability matrix *K* was constructed according to Equation 10, as shown in Figure 4. The skeleton matrix *S* was derived using Equation 11, which is illustrated in Figure 5. Based on Equation 12 through Equation 16, the hierarchical division was completed, as presented in Table 3. Finally, the ISM hierarchical structure model was visualized by integrating the skeleton matrix and the hierarchical results, as shown in Figure 6.

**Table 3:** Decomposed structure.

***X_*i*_***	***R*(*s_*i*_*)**	***A*(*s_*i*_*)**	***C*(*s_*i*_*)**
1	1, 5, 6, 7, 8, 9, 10, 11, 12, 13, 14, 15, 16	1	1
2	2, 5, 6, 7, 8, 9, 10, 11, 12, 13, 14, 15, 16	2	2
3	3, 5, 6, 9	3	3
4	4, 5	4	4
5	5	1, 2, 3, 4, 5, 7, 9, 10, 11, 12, 15, 17	5
6	6	1, 2, 3, 6, 7	6
7	5, 6, 7, 9	1, 2, 7	7
8	8	1, 2, 8, 11, 17	8
9	5, 9	1, 2, 3, 7, 9, 10, 11, 12, 15, 17	9
10	5, 9, 10	1, 2, 10	10
11	5, 8, 9, 11	1, 2, 11	11
12	5, 9, 12	1, 2, 12	12
13	13	1, 2, 13	13

In the cultural, ecological, and economic system of bridge-tourism integration, understanding the hierarchical relationships among the elements is crucial. The DEMATEL analysis identified key top-level driving factors, such as historical background (*X*_1_) and architectural art (*X*_2_), and confirmed their central roles within the system. The historical background (*X*_1_) embodies the cultural heritage and historical significance of bridges, serving as the foundation for bridge-tourism integration initiatives. Architectural art (*X*_2_) contributes to visual and aesthetic appeal, which attracts tourists. These top-level driving factors exert substantial influence on mid-level and lower-level elements through hierarchical transmission, as shown in Figure 6. Mid-level factors, including cultural activities (*X*_3_), geographic location (*X*_7_), infrastructure (*X*_11_), and social culture (*X*_10_), function as mediators within the system. These factors enhance the coordinated operation of other elements, operating under the influence of the top-level drivers. For example, cultural activities (*X*_3_) and cultural heritage (*X*_15_) augment the attractiveness and experiential value of projects by fostering deeper visitor engagement. Lower-tier factors, such as ecosystem (*X*_8_) and policy regulations (*X*_9_), bridge the mid-level and base-level factors by providing ecological sustainability and institutional support, thus ensuring the long-term operation and sustainability of the system. Base-level outcomes, including tourist satisfaction (*X*_13_), economic benefits (*X*_14_), and ecological education (*X*_8_), directly reflect the final performance of bridge-tourism integration. These factors provide feedback to the mid-level and top-level elements, completing the causal chain within the system.

### 5.3 MICMAC calculation and analysis

**Figure 7:**
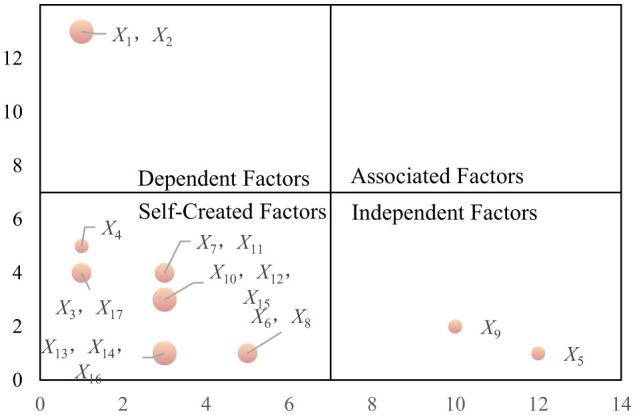
Driving force-dependency matrix.

Through MICMAC analysis, the driving force and dependence of each factor within the system were assessed. By calculating the sum of the rows and columns of the reachability matrix *K*, the driving force *P* and dependence *J* for each factor were derived. The driving force *P* reflects the extent to which a factor influences others, while dependence *J* indicates the degree to which a factor is influenced by other elements within the system. The results of the analysis are presented in Table 4 and Figure 7.

**Table 4:** Driving force-dependency value table.

**Factors**	***P_*i*_***	***J_*i*_***	**Factors**	***P_*i*_***	***J_*i*_***	**Factors**	***P_*i*_***	***J_*i*_***
*X*_1_	1	13	*X*_7_	3	4	*X*_13_	3	1
*X*_2_	1	13	*X*_8_	5	1	*X*_14_	3	1
*X*_3_	1	4	*X*_9_	10	2	*X*_15_	3	3
*X*_4_	1	5	*X*_10_	3	3	*X*_16_	3	1
*X*_5_	12	1	*X*_11_	3	4	*X*_17_	1	4
*X*_6_	5	1	*X*_12_	3	3			

The driving force-dependence matrix analysis revealed that core driving factors, such as historical background (*X*_1_) and architectural art (*X*_2_), exhibit both high driving force and high dependence. These factors not only play a pivotal role in shaping the overall structure of the system but also depend on the collaboration of supporting factors, such as policy regulations (*X*_9_) and infrastructure (*X*_11_). In contrast, independent factors, such as environmental friendliness (*X*_5_), demonstrate high driving force but low dependence, thereby contributing to the system's stability. The MICMAC analysis identified no strongly interdependent factors, suggesting a well-defined hierarchical structure within the bridge-tourism integration system. This clear structure helps to minimize the potential instability that could arise from excessive interdependence among factors.

### 5.4 Overall performance of bridge-tourism integration

The overall performance of bridge-tourism integration is evaluated not only through economic benefits but also by its contributions to cultural preservation and ecological sustainability. A comprehensive assessment of its multi-dimensional value—spanning cultural, ecological, and economic aspects—offers a holistic understanding of the system's integrated performance. In the cultural dimension, bridge-tourism integration provides visitors with enriching cultural experiences, grounded in the bridge's historical significance and architectural value. Core driving factors, such as the historical background and architectural art, enhance cultural activities (*X*_3_) and cultural heritage (*X*_15_), hence, fostering greater cultural recognition and engagement among tourists. In the ecological dimension, bridge-tourism integration emphasizes environmental protection and sustainable practices. Through the implementation of environmentally friendly measures (*X*_5_) and the use of sustainable technologies (*X*_6_), the negative ecological impacts associated with tourism are minimized, ensuring long-term ecological health and sustainability. In the economic dimension, bridge-tourism integration projects generate substantial economic benefits for local communities. These projects stimulate growth in related sectors by increasing tourist arrivals, and spending levels, and creating employment opportunities. Consequently, bridge-tourism integration not only drives the development of local industries but also fosters broader regional economic growth.

## 6 Discussion

### 6.1 Deep integration of culture and ecology

Achieving deep integration between cultural and ecological values is a key strategy for advancing the high-quality development of bridge-tourism integration. The convergence of cultural heritage and ecological conservation not only enriches the tourism experience but also reinforces environmental sustainability. Firstly, the preservation and interpretation of the historical and cultural significance of bridges should be prioritized to ensure the continuity of cultural value. Interactive initiatives such as guided cultural tours, historical storytelling sessions, and art exhibitions can enhance visitors‘ cultural recognition and provide immersive experiences that highlight the uniqueness and symbolic meaning of bridges. For bridges with notable historical significance, protective measures—such as visitor flow management, access regulation, and maintenance protocols—should be implemented to prevent degradation caused by excessive tourist activity and to preserve their cultural integrity over time. Secondly, the incorporation of eco-friendly technologies into bridge construction and maintenance processes should be promoted, aligning environmental protection with cultural engagement. The use of green technologies and sustainable materials—such as biodegradable composites and energy-efficient systems—can mitigate the environmental impact of bridge infrastructure. Furthermore, bridges may serve as ecological education hubs, with the installation of educational displays in scenic areas to inform visitors about sustainable construction practices and environmental stewardship, thereby enhancing public awareness and pro-environmental behavior. Thirdly, cultural activities should be innovatively designed to reflect and reinforce the ecological and cultural significance of bridges. Events such as eco-hiking excursions, bridge photography competitions, and cultural-ecological festivals can attract tourists to explore the aesthetic and environmental dimensions of bridges. Themed experiences—such as “Bridge Ecology Tours” or “Cultural-Ecological Art Exhibitions”—can further deepen visitors' understanding of the interdependence between cultural heritage and ecological systems. Enriching and engaging cultural programming fosters active participation and emotional connection, thereby advancing the integrated development of cultural and ecological tourism under the bridge-tourism model.

### 6.2 Creating a harmonious environment of nature and humanity

In the process of advancing high-quality development in bridge-tourism integration, the coordinated interaction between natural and human environments serves as a fundamental basis for building a sustainable tourism system. However, previous studies have highlighted persistent gaps between ecological conservation and local development, particularly in the form of regulatory enforcement inconsistencies and conflicting stakeholder interests, which may undermine the effectiveness of policy implementation. Therefore, fostering a harmonious environment requires the integrated coordination of ecological protection mechanisms, institutional support frameworks, and community-based governance systems to enhance systemic resilience. First, ecological monitoring should not be limited to technological deployment but should be embedded within a full life-cycle management framework. In addition to applying modern tools—such as sensors and unmanned aerial vehicles (UAVs)—for real-time environmental data collection, it is imperative to establish cross-sectoral data-sharing platforms. These platforms would enable closed-loop governance by facilitating seamless transitions from monitoring to early warning and intervention, thereby improving the responsiveness and effectiveness of ecological regulation. Second, a well-developed policy and regulatory system forms the institutional foundation for the successful implementation of bridge-tourism integration. Nevertheless, policy efficacy often depends on the degree of alignment between regulatory rigidity and local contextual flexibility. To address this, it is recommended that localized policies adopt a “baseline constraint + adaptive guidance” approach—setting firm boundaries for ecological and cultural protection while allowing for region-specific policy adjustments. The regulatory system should also incorporate mechanisms such as performance-based evaluations, public participation, and iterative feedback from case studies to support dynamic optimization and continuous refinement. Third, promoting meaningful community participation is not only essential for cultivating a humanistic environment but also entails structural shifts in local governance. Studies have shown that community engagement—if not institutionalized and capacity-supported—tends to be symbolic rather than substantive. Therefore, a multi-stakeholder governance model involving communities, governments, and market actors should be established. This framework should define clear boundaries of rights and responsibilities for community actors, develop incentive schemes, and implement technical training programs to enhance the actual agency of local communities in the management and co-governance of bridge-tourism initiatives.

### 6.3 Optimizing tourist experience and economic benefits

The high-quality development of bridge-tourism integration relies not only on enhancing visitor satisfaction but, more critically, on the effective redistribution of tourism-generated revenues to local communities. Prior studies have emphasized that opaque benefit-sharing mechanisms or insufficient community involvement often lead to perceptions of inequality at the grassroots level, which in turn diminishes willingness to cooperate and undermines cultural stewardship efforts. First, in improving visitor experience, emphasis should be placed on fostering “emotionally resonant” service environments rather than simply expanding infrastructure. While digital tools such as smart guide systems and barrier-free facilities can enhance convenience, equal attention must be paid to their ability to support cultural immersion and emotional connection. It is recommended that the historical narratives, ecological values, and local stories associated with bridges be integrated into cohesive interpretive frameworks. By leveraging interactive and contextualized experiences, tourism initiatives can evoke deeper affective responses and cultural resonance among visitors. Second, regarding the local return of tourism revenues, rigid profit-sharing or subsidy schemes should be avoided in favor of dynamic benefit-negotiation platforms. While maintaining a guaranteed baseline of community income, mechanisms should be established to encourage community engagement across multiple domains—such as cultural interpretation, homestay operations, and environmental stewardship. Performance-based evaluations, point-based incentive systems, and flexible role allocations can promote a shift from “passive reception” to “proactive participation,” aligning income distribution with responsibilities assumed. Third, it is essential to account for intra-community differentiation in tourism governance and operational participation. Community members differ in capacity, access to resources, and willingness to participate, making uniform policies potentially counterproductive—overburdening the most engaged while marginalizing less involved actors. A tiered and category-based support framework should therefore be developed, providing tailored training, resources, and operational models to match the diverse needs of community subgroups. Such an approach can foster a cooperative ecosystem characterized by functional complementarity and “pluralistic co-existence”.

## 7 Conclusion

This study, through multi-level system analysis and theoretical modeling, comprehensively investigates the complex interactions among factors in the bridge-tourism integration system and evaluates its multi-dimensional value across cultural, ecological, and economic dimensions. The proposed system model categorizes influencing factors into four layers: the driving layer, the middle bridging layer, the connection layer, and the result layer. Using DEMATEL and ISM models, the roles and interconnections of these factors within the system were systematically analyzed. The findings confirm that historical background (*X*_1_) and architectural art (*X*_2_), as top-level driving factors, play a pivotal role in the bridge-tourism integration system. These factors directly enhance cultural experiences and tourist satisfaction (*X*_13_). Middle bridging factors, including cultural activities (*X*_3_), geographical location (*X*_7_), infrastructure (*X*_11_), social culture (*X*_10_), tourism experience (*X*_12_), economic benefits (*X*_15_), and awareness of sustainable development (*X*_17_), act as crucial mediators, linking core driving forces with other system elements. This interaction enhances the system's effectiveness by promoting synergy among its components. Lower-level connection factors, such as policy regulations (*X*_9_) and environmental friendliness (*X*_4_), ensure the stability and sustainability of the system by providing regulatory and ecological constraints. These factors regulate the functioning of the core and bridging layers, reinforcing the sustainable development of bridge-tourism integration. At the base level, result factors—including sustainable technologies (*X*_6_), ecological education (*X*_8_), tourist satisfaction (*X*_13_), economic benefits (*X*_14_), and management level (*X*_16_)—represent direct system outcomes. These outcomes serve as key performance indicators across cultural, ecological, and economic dimensions, reflecting the success of the system's operations. Moreover, through feedback mechanisms, result factors influence driving forces and other system components, promoting self-regulation and continuous optimization. The hierarchical structure revealed by this analysis highlights the inherent stability and functionality of the bridge-tourism integration system, demonstrating its capacity to achieve multi-dimensional value. Independent factors, such as policy regulations (*X*_9_) and infrastructure (*X*_5_), exhibit strong driving power, shaping the system's structure and functionality. Dependent factors, including historical background (*X*_1_) and architectural art (*X*_2_), leverage this support to promote cultural dissemination and ecological preservation. MICMAC analysis further clarifies the positions and interdependencies of these factors, offering a robust theoretical foundation for understanding the complexity of bridge-tourism integration. From a holistic perspective, bridge-tourism integration generates significant integrated benefits across cultural, ecological, and economic dimensions. This study demonstrates that bridge-tourism integration serves as an effective mechanism for transmitting cultural heritage, a practical platform for ecological conservation, and a driving force for regional economic growth. By developing a multi-dimensional performance evaluation system, this research highlights the comprehensive value of bridge-tourism integration and provides critical theoretical and practical guidance for the future management and development of tourism initiatives.

## Data Availability

The original contributions presented in the study are included in the article/Supplementary material, further inquiries can be directed to the corresponding author.
