# Supplementary material for: Evaluating the multi-dimensional values of bridge-tourism integration: an empirical study using the DEMATEL—ISM—MICMAC method
Source: Front Public Health. 2025 Jul 17;13:1566420. doi: 10.3389/fpubh.2025.1566420 (PMC12310712; doi:10.3389/fpubh.2025.1566420)
Supplement: Supplementary file 1 [file Data_Sheet_1.docx]

**Appendix A: Interview Outline on the Influencing Factors of Bridge-Tourism Integration Performance**

Dear Participant,

Thank you for taking part in this expert consultation on the **influencing factors of multidimensional performance in bridge-tourism integration**. This study is jointly conducted by academic and research institutions and aims to identify and structurally analyze the cultural, ecological, and economic dimensions of bridge-tourism integration using a combined system approach. The survey is anonymous, and all data will be used strictly for academic purposes.

This study focuses on the following core question: *What factors influence the performance of bridges as cultural carriers, ecological nodes, and economic enablers in tourism contexts?* How do these factors interact, and what is their causal and hierarchical relationship? The present survey combines expert interviews with quantitative scoring to generate foundational data for model construction. Your expertise will make a significant contribution to this research.

**Part I: Background Information of the Interviewee**

1. Your age group:（ ）

| A. 20–30 | B. 31–40 | C. 41–50 | D. 51 and above |
| --- | --- | --- | --- |

1. Your gender:

| A. Male | B. Female |
| --- | --- |

1. Your field of work:

| A. Bridge Engineering | B. Cultural Tourism | C. Environmental Protection |
| --- | --- | --- |
| D. Materials/Chemical Engineering | E. Policy and Public Administration | F. Other: _______ |

1. Your current role:

| A. University Faculty/Researcher | B. Government Officer | C. Industry Professional |
| --- | --- | --- |
| D. Graduate Student | E. Other |  |

1. Are you actively involved in or regularly following bridge-tourism integration projects?

| A.Yes | B.No |
| --- | --- |

**Part II: Interview Questions**

1. In your opinion, what are the main cultural, ecological, or economic values embodied by bridges in tourism development? Please provide examples where possible.
2. Based on your experience or observations, what are the notable achievements and challenges in bridge-tourism integration projects (e.g., tourist feedback, ecological protection, cultural representation, economic benefits)?
3. In your professional judgment, which factors are most critical in determining the performance of bridge-tourism integration? For example: geographical location, cultural activities, ecological friendliness, policy support, visitor satisfaction, etc.
4. Do you believe there is a causal or hierarchical relationship among these factors? Please briefly explain your reasoning or how you would conceptually group or rank them.
5. For current bridge-tourism initiatives, what aspects do you believe should be prioritized for improvement—cultural preservation, ecological technologies, or tourism experience?
6. What are the key limitations or challenges in scientifically evaluating bridge-tourism integration performance, based on your observation or experience?
7. What is your perspective on the role of local communities in bridge-tourism projects? Are communities actively involved and benefiting economically or culturally?
8. Do you have any other suggestions or insights that you believe should be considered or explored further in this study?
9. If you are open to follow-up contact for model validation or further consultation, please leave your contact information (used strictly for research purposes):

**Appendix B: Expert Evaluation Questionnaire on the Relationship between Influencing Factors of Bridge-Tourism Integration**

Dear Expert,

Greetings! I am currently conducting a study on the influencing factors of bridge-tourism integration performance. This questionnaire is designed for the quantitative analysis stage of the research, aiming to determine the interrelationships and degrees of influence among various factors impacting the effectiveness of bridge-tourism integration. Your professional evaluation and insights are critical to this study.

Please rest assured that all data collected will be used exclusively for academic purposes and handled anonymously. Kindly rate the degree of influence between the following 18 factors based on your expertise using a five-point Likert scale:
**No Influence = 0, Very Weak Influence = 1, Weak Influence = 2, Strong Influence = 3, Very Strong Influence = 4.**

We sincerely appreciate your time and support!

Table 1. Conceptual Connotation of Influencing Factors

| **Major Category** | **Secondary Category** | **Influencing Factor** | **Description** |
| --- | --- | --- | --- |
| **Driving Dimension** | Cultural Factors | Historical Background (*X*_1_) | Historical origins and cultural significance of the bridge |
|  |  | Architectural Art (*X*_2_) | Aesthetic and engineering design of the bridge |
|  |  | Cultural Activities (*X*_3_) | Cultural events held on or around the bridge |
|  | Ecological Factors | Environmental Friendliness (*X*_4_) | Environmental impact of the bridge's construction and operation |
|  |  | Ecological Education (*X*_5_) | Role of the bridge in promoting environmental awareness |
|  |  | Sustainable Technologies (*X*_6_) | Use of eco-friendly materials and construction techniques |
| **Environmental Dimension** | Natural Environment | Geographical Location (*X*_7_) | Location and landscape characteristics of the bridge |
|  |  | Ecosystem (*X*_8_) | Integrity and biodiversity of the surrounding ecosystem |
|  | Human Environment | Policy and Regulations (*X*_9_) | Relevant policies and regulations supporting bridge-tourism integration |
|  |  | Social Culture (*X*_10_) | Cultural atmosphere and local community involvement |
|  |  | Infrastructure (*X*_11_) | Transportation and service facilities supporting bridge-tourism integration |
| **Carrying Dimension** | Tourists | Tourism Experience (*X*_12_) | Visitors' engagement and experiences within bridge-tourism integration |
|  |  | Satisfaction (*X*_13_) | Recognition of cultural and ecological values by tourists |
|  | Local Communities | Economic Benefit (*X*_14_) | Economic benefits from bridge-tourism integration for the local community |
|  |  | Cultural Heritage (*X*_15_) | Local community participation in cultural activities and preservation |
|  | Managers | Management Level (*X*_16_) | Planning and maintenance of bridge-tourism integration resources |
|  |  | Sustainable Development Awareness (*X*_17_) | Awareness of ecological and cultural protection in management |

**Section II: Basic Information Survey**

1. Your age group:（ ）

| A. 20–30 | B. 31–40 | C. 41–50 | D. 51 and above |
| --- | --- | --- | --- |

1. Your gender:

| A. Male | B. Female |
| --- | --- |

1. Your field of work:

| A. Bridge Engineering | B. Cultural Tourism | C. Environmental Protection |
| --- | --- | --- |
| D. Materials/Chemical Engineering | E. Policy and Public Administration | F. Other: _______ |

1. Your current role:

| A. University Faculty/Researcher | B. Government Officer | C. Industry Professional |
| --- | --- | --- |
| D. Graduate Student | E. Other |  |

1. Are you actively involved in or regularly following bridge-tourism integration projects?

| A.Yes | B.No |
| --- | --- |

**Section III: Evaluation of the Relationships Between Influencing Factors**
*Please refer to Table 2 below for the scoring of inter-factor influence.*

**Table 2. Scoring Matrix for Interrelationships Between Influencing Factors**
(*To be attached as the completed version from Appendix B*)

|  | *X*_1_ | *X_2_* | *X_3_* | *X_4_* | *X_5_* | *X_6_* | *X_7_* | *X_8_* | *X_9_* | *X_10_* | *X_11_* | *X_12_* | *X_13_* | *X_14_* | *X_15_* | *X_16_* | *X_17_* |
| --- | --- | --- | --- | --- | --- | --- | --- | --- | --- | --- | --- | --- | --- | --- | --- | --- | --- |
| *X_1_* | 0 |  |  |  |  |  |  |  |  |  |  |  |  |  |  |  |  |
| *X_2_* |  | 0 |  |  |  |  |  |  |  |  |  |  |  |  |  |  |  |
| *X_3_* |  |  | 0 |  |  |  |  |  |  |  |  |  |  |  |  |  |  |
| *X_4_* |  |  |  | 0 |  |  |  |  |  |  |  |  |  |  |  |  |  |
| *X_5_* |  |  |  |  | 0 |  |  |  |  |  |  |  |  |  |  |  |  |
| *X_6_* |  |  |  |  |  | 0 |  |  |  |  |  |  |  |  |  |  |  |
| *X_7_* |  |  |  |  |  |  | 0 |  |  |  |  |  |  |  |  |  |  |
| *X_8_* |  |  |  |  |  |  |  | 0 |  |  |  |  |  |  |  |  |  |
| *X_9_* |  |  |  |  |  |  |  |  | 0 |  |  |  |  |  |  |  |  |
| *X_10_* |  |  |  |  |  |  |  |  |  | 0 |  |  |  |  |  |  |  |
| *X_11_* |  |  |  |  |  |  |  |  |  |  | 0 |  |  |  |  |  |  |
| *X_12_* |  |  |  |  |  |  |  |  |  |  |  | 0 |  |  |  |  |  |
| *X_13_* |  |  |  |  |  |  |  |  |  |  |  |  | 0 |  |  |  |  |
| *X_14_* |  |  |  |  |  |  |  |  |  |  |  |  |  | 0 |  |  |  |
| *X_15_* |  |  |  |  |  |  |  |  |  |  |  |  |  |  | 0 |  |  |
| *X_16_* |  |  |  |  |  |  |  |  |  |  |  |  |  |  |  | 0 |  |
| *X_17_* |  |  |  |  |  |  |  |  |  |  |  |  |  |  |  |  | 0 |
